# Targeted Regulation and Treatment Application of Liquid–Liquid Phase Separation in Human Diseases

**DOI:** 10.3390/ijms27219662

**Published:** 2026-10-28

**Authors:** Yuanxin Zhang, Qingqing Xu, Yujie Liu, Kejia Yao, Nuo Chen, Guangchao Sui, Dangdang Li, Jian Zheng

**Affiliations:** 1 College of Life Science, Northeast Forestry University, Harbin 150040, China; 2 School of Future Technology, Northeast Forestry University, Harbin 150040, China; 3 Aulin College, Northeast Forestry University, Harbin 150040, China

**Keywords:** liquid–liquid phase separation (LLPS), biomolecular condensates, human diseases, therapeutic targeting

## Abstract

Liquid–liquid phase separation (LLPS) is widely recognized as a fundamental mechanism underlying the formation of membraneless organelles, which are critical for maintaining spatiotemporal precision in cellular function. Dysregulation of LLPS has become increasingly recognized as a key driver underlying the development of multiple human diseases, thereby establishing this process as a rapidly growing focus in biomedical research. In this review, we examine the contributions of LLPS to cellular pathophysiology. We start by outlining the fundamental biophysical and molecular principles that govern LLPS formation. We further explore how aberrant phase transitions contribute to disease mechanisms across different organ systems. Building on this mechanistic framework, we assess current and potential therapeutic strategies aimed at targeting LLPS for clinical intervention. Overall, this review aims to synthesize current understanding of LLPS in disease etiology and to highlight its potential as a novel target for future therapeutic development.

## 1. Introduction

From a physical chemistry perspective, Liquid–liquid phase separation (LLPS) refers to the process by which a homogeneous liquid mixture spontaneously demixes into two immiscible liquid phases. Within biological systems, LLPS is widely regarded as a phenomenon in which intracellular biological macromolecules such as proteins and RNA, undergo intermolecular interactions that lead to an increase in concentration [1]. Upon reaching a certain threshold, these macromolecules can form “membraneless droplets” or “particles”. In a typical binary mixture, LLPS partitions the components into two distinct phases: a dense phase and a dilute phase [2].

The occurrence of LLPS in solution is tightly regulated by multiple factors, including the intrinsic properties of macromolecules, solution conditions, and environmental parameters such as concentration, temperature, and pH [3]. The resulting condensates are highly sensitive to changes in both the intracellular and extracellular environments. Physico-chemically and physiologically, LLPS is stringently regulated, restricting LLPS to a limited subset of cellular components despite the broad capacity of biomolecules to undergo phase separation [3,4]. This observation can be effectively explained by a hierarchical organizational model of cellular composition. In this model, scaffold molecules drive LLPS through multivalent interactions to form condensate cores, which then recruit client molecules to build functional networks for distinct biological tasks [4]. Protein molecules play a crucial role in this process [3].

Proteins capable of forming condensates are generally classified into two categories: those containing intrinsically disordered regions (IDRs), and those possessing multiple folded domains that mediate interactions with other proteins [5,6]. Although these two types of proteins differ in their mechanisms of undergoing LLPS, they share the common feature that LLPS is driven by multivalent interactions, including protein-protein contacts, protein-nucleic-acid interactions, and protein homo-association [7]. Multivalent interactions therefore serve as a central driving force underlying many LLPS systems.

The formation capacity and strength of multivalent interactions are regulated by multiple molecular elements. IDRs lack stable tertiary structures and mediate abundant weak and dynamic molecular contacts such as electrostatic interactions, hydrophobic interactions, π–π stacking and cation–π interactions. Their amino acid composition, charge distribution and sequence length directly determine protein phase-separation propensity and condensate physicochemical properties. Nucleic acids, mainly RNA, exert bidirectional regulatory effects. RNA acts as a molecular scaffold to recruit RNA-binding proteins and strengthen multivalent interaction networks, and the regulatory outcome depends on RNA–protein stoichiometry: moderate RNA promotes condensate assembly, whereas excess RNA disrupts multivalent networks and leads to condensate disassembly. Post-translational modifications (PTMs) including phosphorylation, acetylation and ubiquitination alter the local charge and hydrophobicity of IDR-containing proteins, functioning as molecular switches to dynamically tune the driving force of phase separation. The biological effects of these modifications are protein- and site-dependent; they can promote or suppress condensate assembly, alter condensate fluidity, and even drive liquid-to-solid phase transition.

**Figure 1:**
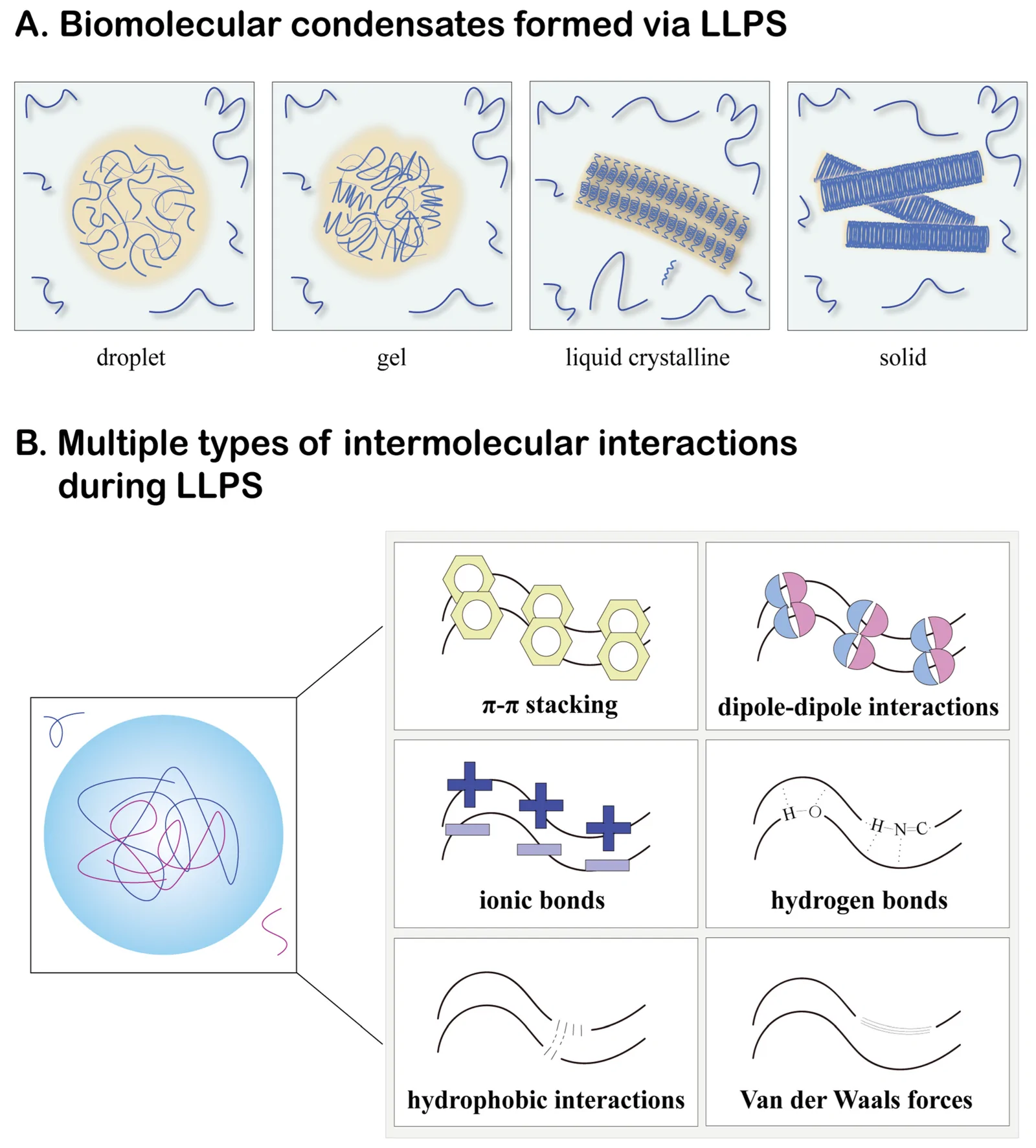
Diverse material states and molecular drivers of biomolecular condensates. (**A**). Biomolecular condensates formed via LLPS initially adopt liquid-like states but can further develop diverse material properties through continuous maturation. As illustrated (from left to right), one canonical chronological trajectory of condensate maturation comprises liquid droplets, gel, liquid-crystalline, and solid condensates, with molecular mobility progressively declining along this sequence. Notably, these non-liquid rigid states primarily result from the aging and maturation of initial liquid condensates, rather than originating directly from LLPS. In addition, solid-state assemblies can also arise through independent parallel thermodynamic pathways such as liquid-solid phase separation (LSPS) or classical direct nucleation from solution, without traversing liquid-droplet intermediates. (**B**). Multiple types of intermolecular interactions can drive the LLPS of proteins. As illustrated (from top to bottom, left to right), these primarily include π–π stacking, dipole–dipole interactions, ionic bonds, hydrogen bonds, hydrophobic interactions, and Van der Waals forces, among others. Biomolecular condensates driven by different dominant interactions may exhibit distinct physicochemical properties and biological functions.

Biomolecular condensates formed via LLPS typically exhibit droplet-like states. However, they can also adopt a spectrum of material properties in cells, including gel, liquid-crystalline, and solid phases [3,8,9] (Figure 1A). This diversity in material properties is not random but is primarily governed by the nature and strength of the intermolecular forces driving LLPS, such as π-π stacking [10], dipole–dipole interactions [11], ionic bonds [12], hydrogen bonds [10], hydrophobic interactions [8], and Van der Waals forces [8] (Figure 1B). The specific balance of these interactions directly influences the structural features of condensates, including their internal fluidity, spatial organization, and viscoelasticity [9]. Strong interactions, such as ionic interactions, frequently promote the formation of gel condensates characterized by fixed three-dimensional networks and reduced molecular mobility [9]. Importantly, ionic interactions can also sustain highly dynamic liquid-state condensates in many biological contexts [13]. In contrast, weaker or transient interactions, such as hydrogen bonding and hydrophobic interactions, can support liquid-crystalline phases, which preserve a degree of molecular order while maintaining fluidity [14]. Notably, these material states are not static; they can undergo reversible or irreversible transitions. For example, Myc-associated zinc finger protein (MAZ) condensates formed by zinc-finger domain shift from a gel to a droplet-like state upon the addition of G-quadruplexes [9]. In addition, phosphorylated tau protein rapidly matures from a droplet state into a gel state [15]. The physiological and pathological significance of condensate material states lies in their direct impact on cellular function. Maintaining normal material properties of condensates is essential for cellular homeostasis, and disruptions to this tightly regulated process frequently drive various diseases, especially neurodegenerative diseases, which involve transitions in the material states of phase-separated condensates. Therefore, understanding the molecular determinants of condensate material states and their dynamics not only elucidates fundamental principles of cellular organization, but also offers a theoretical framework for developing therapeutic strategies aimed at modulating condensate behavior in disease contexts.

**Figure 2:**
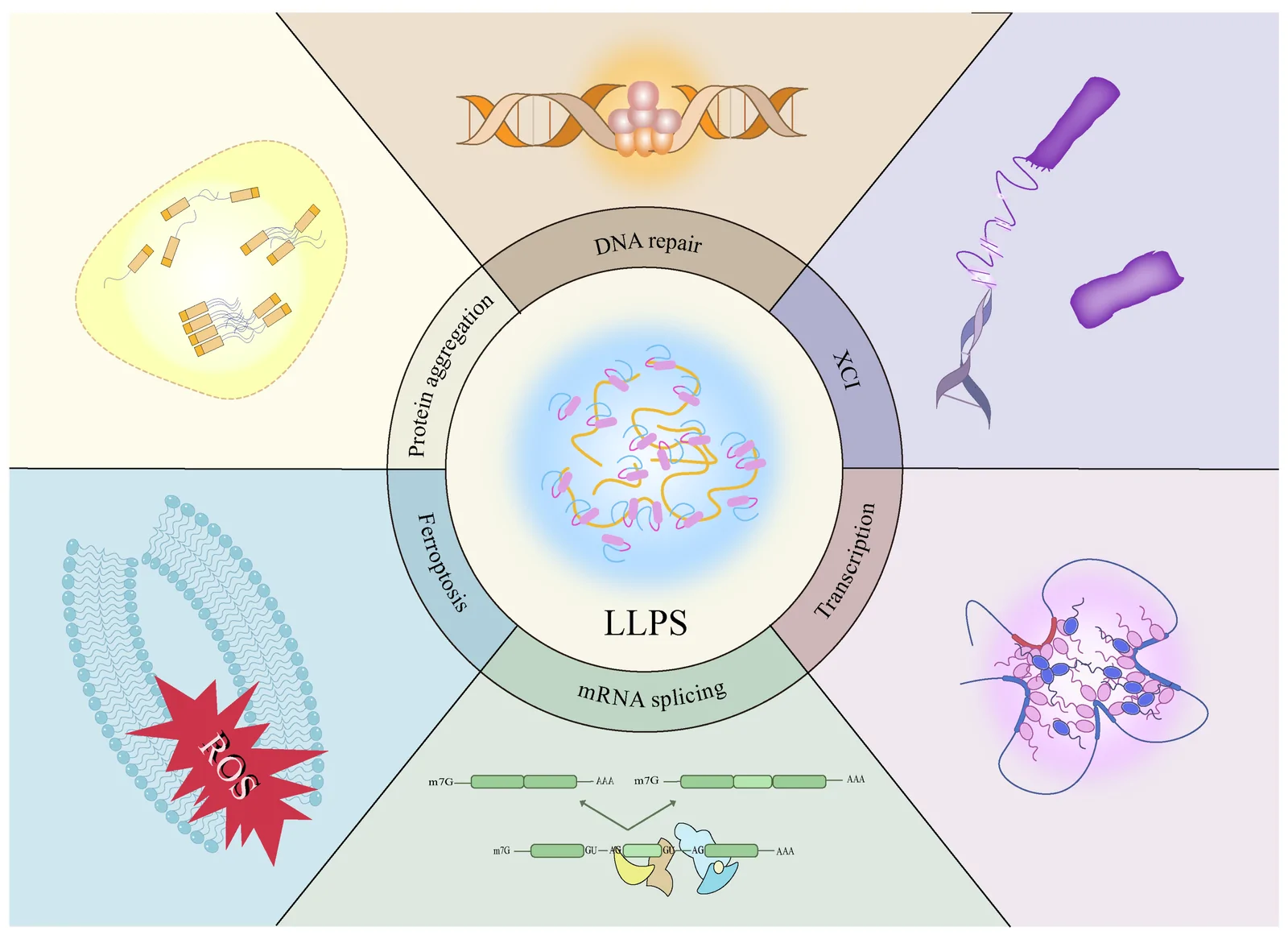
LLPS plays a critical role in various key biological processes. Through the formation of membraneless biomolecular condensates, LLPS is involved in transcription regulation, alternative splicing, ferroptosis, DNA damage repair, and protein aggregation, contributing significantly to the maintenance of cellular homeostasis.

In recent years, LLPS has been extensively studied to characterize the structural organization mechanisms of membraneless organelles [16,17,18]. Unlike classical membrane-bound organelles, the condensates display both high dynamicity and compositional flexibility. These condensates function as hubs that concentrate specific biological macromolecules, allowing for efficient biochemical reactions and a rapid, low-energy response to changes in the external environment. This tunability enables condensates to undergo dynamic material exchange with their surroundings and, in some cases, transition between material states (e.g., from liquid-like to gel-like), which is critical for their biological functions and responses to environmental changes [18]. Beyond organizing intracellular biochemistry, LLPS is involved in a wide range of cellular activities and physiological processes. Research has linked LLPS to transcription [19], ferroptosis [20], DNA damage and repair [21], X chromosome inactivation (XCI) [22], protein aggregation [23], and mRNA splicing [16] through the formation of membraneless condensates (Figure 2), underscoring its broad functional relevance. Because of its central role in cellular organization and signaling, the misregulation of LLPS—whether through aberrant condensation, aging, pathological modifications, or environmental stress—is increasingly associated with disease states, including neurodegenerative disorders and cancer [24].

As discussed above, the dysregulation of LLPS is intricately associated with the pathogenesis and progression of diverse human diseases. Building upon the current body of research, this review systematically consolidates the spectrum of LLPS-associated pathologies and elucidates the underlying molecular regulatory mechanisms operating under pathological conditions. Specifically, we highlight emerging research hotspots in the field, with particular emphasis on the role of stress granules in neurodegenerative disorders. Furthermore, this article comprehensively integrates and summarizes potential therapeutic strategies targeting LLPS, complementing existing review literature in this research area.

## 2. Common Human Diseases Associated with LLPS

LLPS sustains the precise spatiotemporal organization of critical cellular processes, whereas its dysregulation or aberrant phase transitions have emerged as primary drivers underlying the development and progression of a broad range of human diseases [25].

### 2.1. LLPS and Neurodegenerative Diseases

LLPS has emerged as a pivotal biophysical mechanism driving the formation of biomolecular condensates, which sustain essential cellular functions under physiological conditions. However, under pathological conditions, this same process can mediate the aggregation of aberrant proteins, which represents the core pathological hallmark of multiple neurodegenerative diseases, including Amyotrophic Lateral Sclerosis (ALS), Alzheimer’s Disease (AD), and limbic-predominant age-related TDP-43 encephalopathy (LATE) [26,27,28]. A growing body of evidence suggests that LLPS plays a dominant role in the pathological transition of proteins such as FUS, tau, and TDP-43 from dynamic liquid droplets to irreversible amyloid aggregates [29]. This process, often referred to as “maturation” or “liquid-to-solid transition”, is influenced by aging, disease-related mutations, and post-translational modifications (PTMs), which collectively alter the material properties of protein condensates [23] (Table 1).

Protein aggregation in patient tissues is tightly modulated by disease-associated mutations and PTMs [8,25]. In proteins harboring disease-related mutations, the propensity for LLPS is enhanced, which potentially accelerates the formation of pathogenic protein aggregates [15]. PTMs can influence protein charge, conformation, and interactions with other molecules [23], thereby affecting the key interaction forces that govern LLPS. However, the precise effects of many mutations and PTMs on phase behavior remain incompletely understood; therefore, it is essential to focus on studying the impact of pathological mutations and PTMs on proteins to facilitate the development of new therapeutic drugs [25]. In AD and related tauopathies, hyperphosphorylated tau undergoes LLPS to form condensates that rapidly transition into gel states within minutes, before subsequently maturing into insoluble aggregates [15]. This process is exacerbated by disease-specific mutations and PTMs, which strengthen the multivalent interactions underlying aberrant LLPS [8,25]. Similarly, as a key protein in ALS and LATE [41,42,43], TDP-43 can undergo LLPS in vitro and in vivo. Under disease conditions, however, sustained cytoplasmic LLPS promotes the formation of toxic inclusions [44]. Even in the absence of overt aggregation, persistent LLPS of TDP-43 disrupts RNA processing and cellular homeostasis, thereby contributing to neurodegeneration [45,46]. Notably, factors such as changes in isoelectric point and charge distribution further modulate TDP-43 LLPS, highlighting the sensitivity of phase behavior to biochemical alterations [47].

Notably, for protein aggregation driven by liquid–liquid phase separation, this is not an instantaneous event but a multistep process encompassing initial droplet formation, transition to a gel state, and eventual maturation into amyloid fibrils. However, the precise alterations in protein conformation and interaction forces underlying such phase transitions remain to be fully elucidated. There is a notable deficiency in the understanding of the relationship between different types of protein aggregates. Despite these advances, key questions remain unanswered. Through which potential mechanisms does LLPS play a pathological role in these diseases? Additionally, how can the application of targeted inhibitors rationally optimized to prevent the disruption of the physiological functions of normal condensates while maintaining preserving protein concentration homeostasis? Unraveling the molecular drivers of aberrant LLPS will enable the development of effective strategies to restore proteostasis and alleviate disease progression.

### 2.2. LLPS and Cancer

Cancer initiation and progression arise from the disruption of cellular homeostasis and subsequent uncontrolled malignant proliferation [21]. Accumulating evidence indicates that numerous cancer-related proteins participate in transcriptional regulation, translation, signal transduction and protein degradation via LLPS [48]. Aberrant LLPS is extensively implicated in the onset and advancement of various malignancies, including breast cancer and lung cancer [49,50].

A key driver of LLPS in oncogenesis is the presence of IDRs within proteins. These flexible domains, lacking a stable three-dimensional structure, facilitate the multivalent, weak interactions necessary for LLPS. However, imbalances of IDR-mediated interactions may precipitate rapid cancer development. For instance, chromosomal translocations induced by LLPS can lead to the expression of fusion oncoproteins, which may result in tumorigenesis [51]. Notably, approximately 67% of these fusion oncogenes are capable of forming such abnormal LLPS condensates [52]. Furthermore, the dysregulation of IDRs can disrupt critical cellular processes, including alterations in the spatial organization of molecules within cells, improper transport mechanisms, disturbances in the intracellular environment, and dysregulation of gene expression [53]. The precise mechanisms by which pathological IDR mutations disrupt LLPS and contribute to oncogenesis remain an active area of investigation.

Importantly, oncogenic LLPS driven by fusion proteins is closely intertwined with extrachromosomal DNA (ecDNA)-mediated transcriptional hyperactivation, representing a unique tumor-specific phase-separation mechanism [51,52]. Focal oncogene amplifications and driver fusion genes frequently localize to circular ecDNA elements, which recruit epigenetic readers such as BRD4 to assemble large-scale phase-separated transcriptional clusters. These condensates further form in-trans associated contact domains (I-TADs), which override canonical chromatin regulation and drive robust oncogene overexpression to sustain malignant proliferation [51,52]. Distinct from physiological LLPS assembled by wild-type proteins, fusion oncoproteins and ecDNA clusters generate tumor-specific neomorphic condensates that are completely absent in healthy cells.

The unique properties of LLPS condensates, particularly their dynamicity and dependence on IDRs, present a promising frontier for cancer therapy. The conformational flexibility of IDRs can engage in multiple instantaneous interactions with other molecules, allowing phase-separated condensates to respond rapidly to changes in both intracellular and extracellular environments. Based on this characteristic, we can design drugs that directly target LLPS condensates.

Regarding the specific mechanisms underlying LLPS function in cancer, we provide a detailed discussion in Section 3.

This strategy offers a path to target proteins previously considered “undruggable” due to a lack of conventional binding pockets. Notably, there exists a fundamental therapeutic distinction between LLPS-targeted strategies for cancer and neurodegenerative diseases. As discussed in the neurodegeneration section, targeting wild-type protein phase separation via upstream kinase or phosphatase modulation suffers from severe systemic toxicity and poor selectivity, owing to the essential homeostatic functions of wild-type LLPS in normal cells. In sharp contrast, tumor-specific condensates formed by oncogenic fusions and ecDNA clusters are exogenous neo-structures exclusive to malignant cells. Targeting the unique physical interaction interfaces of fusion oncoprotein LLPS or inducing ecDNA condensate collapse enables highly selective tumor suppression with minimal damage to physiological LLPS processes in healthy tissues, representing a clinically viable and high-specificity therapeutic avenue. However, several challenges must be overcome to realize the clinical potential of LLPS-targeting cancer therapies, including drug resistance arising from mutations that alter LLPS behavior [53], achieving accurate subcellular distribution of molecules within condensates, and firmly establishing causal links between specific LLPS events and tumorigenesis in different cancer contexts. Addressing these hurdles will be critical for translating our understanding of LLPS into novel, effective cancer treatments.

### 2.3. LLPS and Autoimmune Diseases

LLPS has evolved as an essential regulatory mechanism in immune signaling, and growing evidence suggests its potential involvement in the pathogenesis of autoimmune diseases. Although there is currently no direct evidence linking LLPS to autoimmune diseases, several lines of indirect evidence support this connection. For instance, rheumatoid arthritis (RA), a prototypical autoimmune condition, has been reported to show significantly elevated gene expression levels of LLPS-related proteins in affected patients, implying a possible dysregulation of LLPS in disease contexts [54]. Future investigations exploring the interplay between LLPS and autoimmune diseases may establish a novel framework for therapeutic development against autoimmune disorders. For example, in the systemic lupus erythematosus (SLE) database, the deubiquitinase (DUB) USP8 interacts with the stress granule protein DDX3X and catalyzes its deubiquitination, modulating the cGAS-STING signaling pathway and restraining aberrant type I interferon production [55,56]. Preclinical studies indicate that the small molecule USP8 inhibitor DUB-IN-2 can specifically alleviate cGAS-mediated autoimmune diseases in mouse models, suggesting that targeting pathological condensates may hold promise for treating SLE [57]. Similarly, in RA, bioinformatics analyses reveal that the expression of LLPS-related genes such as *MYC* and *MAP1LC3B* is downregulated during inflammatory progression, implicating LLPS in disease mechanisms and highlighting a need to explore its role in RA pathogenesis and treatment [54].

**Figure 3:**
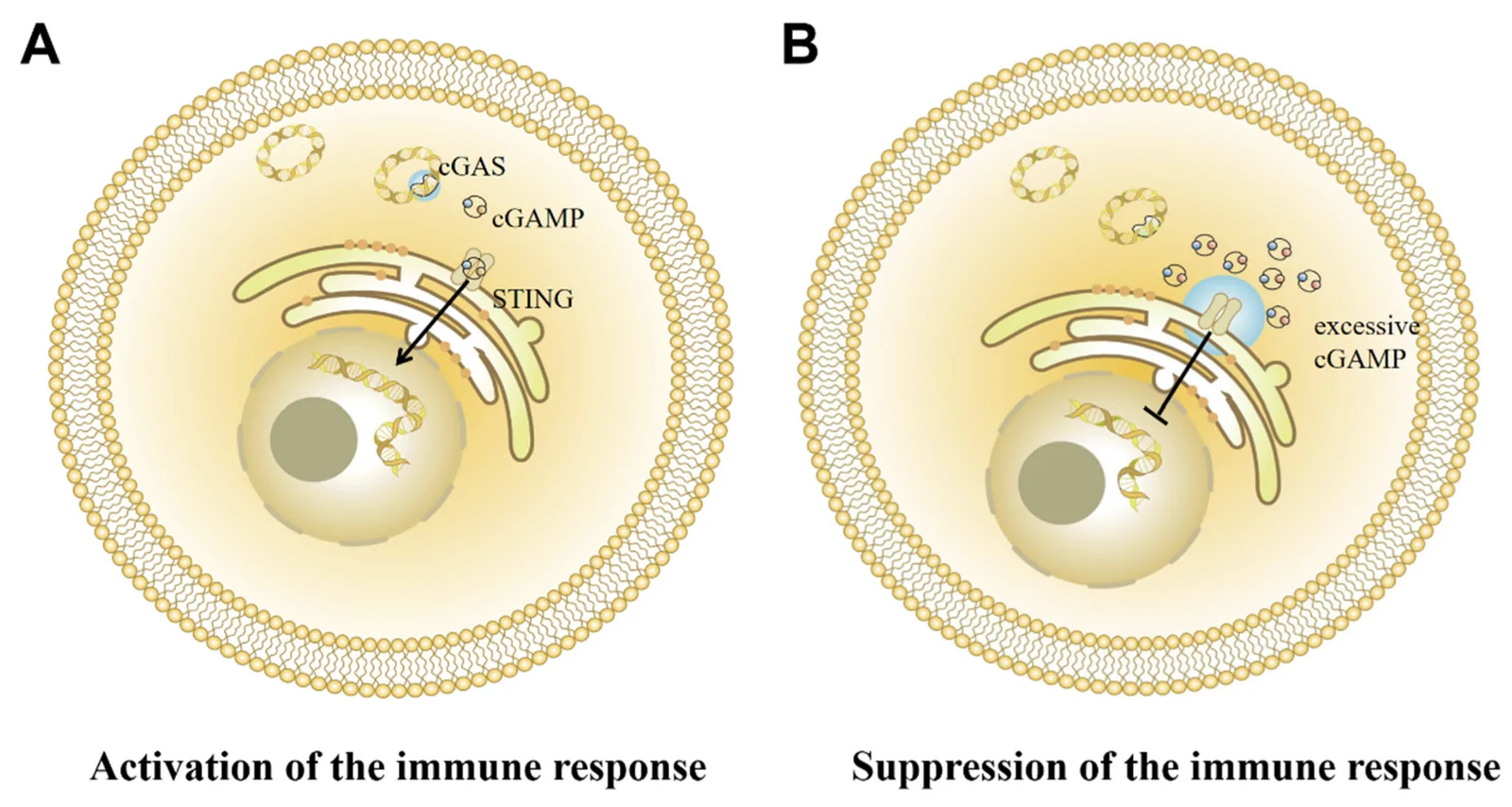
Molecular Mechanisms of cGAS-STING Signaling: (**A**). Immune Activation: Upon entry of exogenous DNA into the cytoplasm, cGAS binds to the DNA, forming a phase-separated condensate that catalyzes the synthesis of the second messenger cGAMP. cGAMP then binds to STING on the endoplasmic reticulum, inducing conformational changes and translocation to the Golgi apparatus. At the Golgi, STING initiates downstream signaling cascades that culminate in the transcription of immune-responsive genes in the nucleus (the transduction events at the Golgi are not shown). (**B**). Immune Suppression: Excessive cGAMP induces LLPS of STING, resulting in the formation of a non-signaling aggregate. This sequesters cGAMP and blocks downstream signal transduction, thereby preventing hyperactivation of innate immunity and maintaining immune homeostasis.

Phase-separated biomolecular condensates play key roles in organizing immune signal transduction. A well-characterized example is the cyclic GMP-AMP synthase-stimulator of interferon genes (cGAS-STING) signaling pathway, where both cGAS and STING proteins can undergo LLPS to facilitate efficient innate immune activation [58]. Upon detecting cytosolic dsDNA, cGAS proteins multivalently interact with DNA to form liquid-like condensates [59,60]. These condensates concentrate cGAS and its substrates, promoting the synthesis of the second messenger cyclic GMP-AMP (cGAMP) and amplifying downstream innate immune signaling [61]. Notably, physiological factors such as free zinc ions can significantly enhance cGAS LLPS, fine-tuning the immune response [59]. Conversely, STING itself can form condensates under sustained cGAMP stimulation, which may serve as a feedback mechanism to suppress excessive signaling, illustrating how LLPS can both activate and regulate immune pathways [61] (Figure 3). Beyond nucleic acid sensing, LLPS participates in RNA-mediated immune responses. For instance, dsRNA can induce the LLPS of the innate sensor NLRP6, leading to inflammasome activation [62]. The protein kinase PKR also forms phase-separated condensates in response to dsRNA [63], highlighting LLPS as a recurrent mechanism across different immune triggers [64]. In addition to nucleic acid sensing and inflammasome activation, LLPS also modulates innate immune defense by regulating autophagic processes against intracellular pathogens.

Beyond innate immunity, LLPS is also critical in adaptive immune signaling, particularly in mediating BCR and TCR signal transduction [65,66]. Upon TCR engagement, downstream signaling proteins such as LAT, GRB2, and SOS1 undergo LLPS to form microclusters that enhance signal transduction efficiency [66]. Similarly, in the B-cell signaling pathway, proteins like SLP65 and CIN85 form phase-separated condensates, frequently associated with vesicles, to prime B-cell activation [66].

LLPS has emerged as a critical biophysical mechanism exploited by pathogens to evade host immune surveillance [67]. By forming biomolecular condensates, viruses and bacteria can dynamically organize their replication machinery and interfere with host defense pathways. For instance, during DNA viral infection, cGAS binds viral DNA and forms phase-separated condensates, thereby initiating innate immune signaling [59]. However, pathogens have evolved countermeasures: *SARS*-CoV-2 disrupts cGAS-mediated condensate formation to suppress interferon production, while enteropathogenic *E. coli* secretes the effector protein NleE, which impairs the ZnF1 domain of the host zinc-finger protein ZRP1, disrupting its LLPS-dependent role in the unfolded protein response and facilitating immune evasion [68]. In the context of viral replication, LLPS plays a particularly critical role in the life cycle of *HIV*-1. As a type of retrovirus, *HIV*-1 relies on a crucial step in the viral replication cycle: the integration of its genomic DNA into the host genome, which has also become a key target in antiretroviral therapy [69]. The integration of viral DNA into the host genome is facilitated by the formation of an LLPS-driven complex between *HIV*-1 integrase and the host cofactor LEDGF/p76 [70]. This phase-separated condensate enhances the efficiency of viral integration by concentrating essential components. Importantly, this mechanism provides a theoretical foundation for the development of new anti-HIV drugs. LLPS provides a unifying biophysical framework for understanding diverse immune-related pathologies, from viral immune evasion to autoimmune dysregulation (Table 2). Deciphering these underlying mechanisms is of great significance for the prevention and treatment of human diseases driven by dysregulated immune responses.

Currently, autoimmune diseases are considered incurable and markedly impair patients’ quality of life. While reports linking LLPS to autoimmune pathology are limited, the established role of LLPS in immune regulation stresses the need for further investigation. Unraveling how LLPS influences autoimmune mechanisms may uncover novel therapeutic strategies, such as modulating the assembly or disassembly of pathogenic condensates to restore immune homeostasis.

### 2.4. LLPS and Cardiovascular Diseases

LLPS has emerged as a significant regulatory mechanism in cardiovascular diseases (CVDs), offering a fresh perspective for understanding their pathogenesis [71]. Uncontrolled assembly of biomolecular condensates formed via LLPS is now recognized as a key factor in disrupting critical cellular processes relevant to cardiovascular health. Recent research into the molecular mechanisms of CVDs have historically concentrated on RNA biology; recent evidence increasingly implicates LLPS as a crucial player [71], which may explain why the molecular mechanism of CVD has not been fully elucidated. Dysregulation of these biomolecular condensates further exacerbates cellular dysfunction and contributes directly to the onset and progression of cardiovascular disorders.

A growing body of evidence suggests that RNA-binding proteins (RBPs), which are pivotal for post-transcriptional regulation, frequently undergo LLPS in the context of CVDs [72]. In such phase-separated systems, long non-coding RNAs (lncRNAs) also play a crucial role, acting as molecular scaffolds to promote the formation and specificity of the droplets [73]. Furthermore, the assembly of SGs via LLPS represents an important cellular response to pathological stimuli [74], such as elevated oxidative stress in overactive cardiomyocytes, and is implicated in the regulatory landscape of CVDs [75]. These findings lay a theoretical foundation for exploring the connection between LLPS and CVD. Aggregation of misfolded proteins, potentially triggered by aberrant LLPS in the setting of disease-associated mutations, represents a well-established mechanism underlying disorders such as dilated cardiomyopathy.

Given that the molecular mechanisms of many CVDs are not fully elucidated, investigating the connection to LLPS provides a promising avenue for research. Exploring how LLPS intersects with established fields like RNA biology could unveil new molecular foundations of cardiovascular pathology and identify innovative therapeutic targets.

## 3. Functional Roles of LLPS in Core Biological Processes

Dysregulation of LLPS has been observed in all the above human diseases [25]. However, LLPS contributes to disease pathogenesis via modulation of a set of fundamental cellular events. In the following section, we summarize the functions of LLPS in core biological processes, including transcriptional regulation, DNA repair, metabolic reprogramming, and innate immunity, as well as pathological dysregulation of immune responses.

### 3.1. LLPS in the Regulation of Gene Transcription

**Figure 4:**
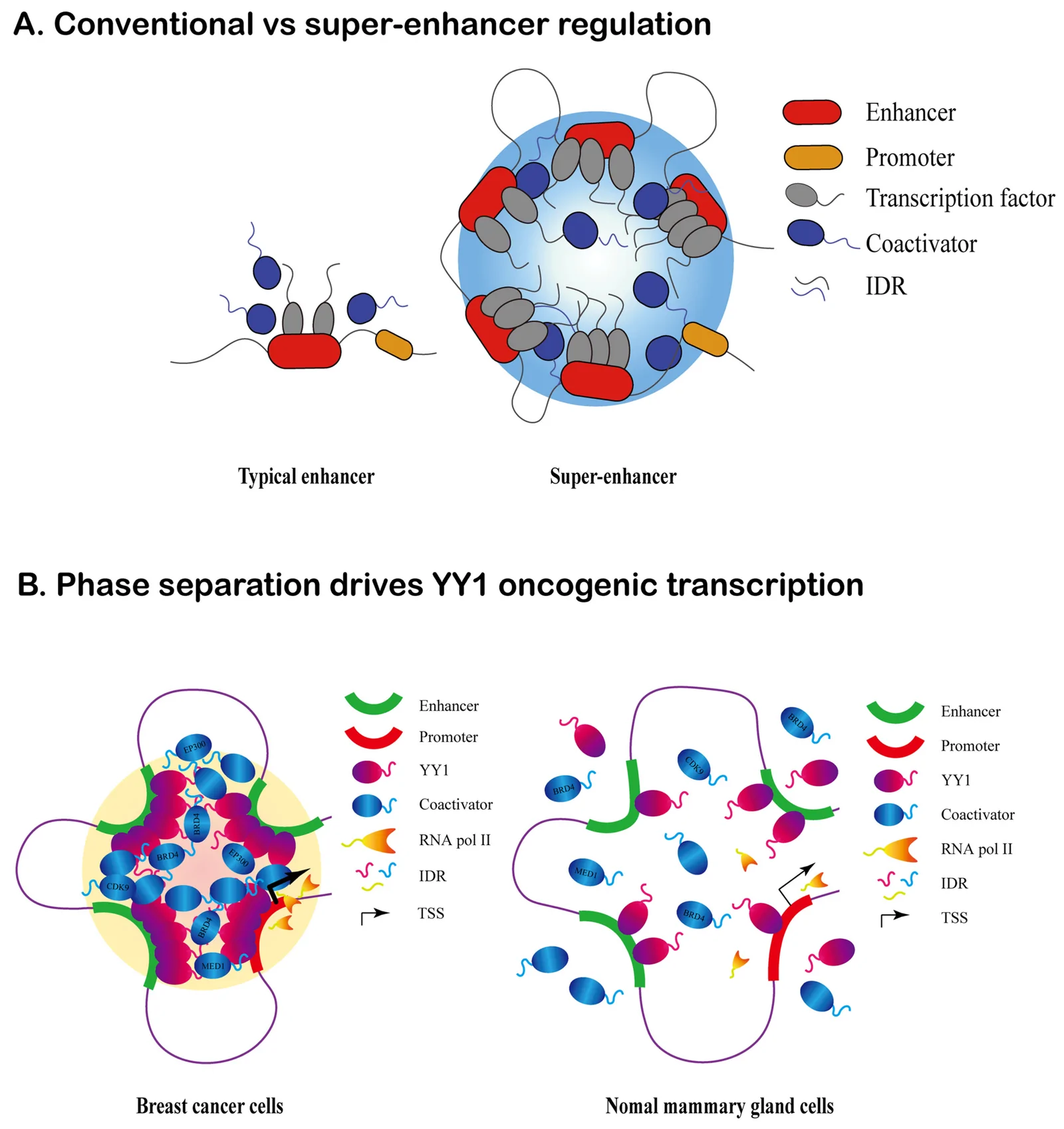
Distinct regulatory mechanisms of typical enhancers and super-enhancers, and YY1-mediated transcriptional control via LLPS in breast cancer: (**A**). Conventional enhancers and super-enhancers operate through distinct transcriptional regulatory mechanisms. The left panel illustrates the typical mode of action of a conventional enhancer: a single enhancer sequence binds specific transcription factors, which then recruit a limited set of coactivators to assemble a basal transcription initiation complex. The right panel depicts the highly efficient mechanism of a super-enhancer: multiple adjacent conventional enhancers cluster spatially to form a cooperative regulatory module. This clustering drives the local enrichment of transcription regulators at high concentrations and promotes the formation of biomolecular condensates via LLPS, thereby enabling the efficient recruitment of large numbers of coactivators and RNA polymerase II, which dramatically amplifies transcriptional activation of downstream genes. (**B**). Schematic diagram illustrating the mechanism by which the transcription factor YY1 regulates transcription through LLPS in breast cancer. In breast cancer cells, YY1 is highly expressed and binds to specific enhancers and promoters, recruiting coactivators to form transcriptional condensates. These condensates exhibit the properties of LLPS, which concentrate RNA polymerase II and facilitate its stable association with promoters, thereby markedly activating downstream gene transcription. In normal breast cells, lower YY1 expression levels are insufficient to drive adequate coactivator recruitment and condensate formation, resulting in minimal transcriptional activity of target genes.

Cells exploit phase-separated condensates to achieve spatiotemporal control over transcriptional regulation. In the context of gene activation, LLPS facilitates the recruitment of transcriptional co-activators to the promoter region [76]. For instance, super-enhancers, highly active regulatory elements, are assembled through the LLPS of transcription factors and co-activators in the nucleus [77] (Figure 4A). Similarly, in breast cancer cells, the transcription factor YY1, which contains IDRs with histidine clusters that drive LLPS, binds to enhancers or super-enhancers to form phase-separated condensates that recruit co-activators like EP300 and BRD4. This promotes the assembly of the transcription initiation complex, including RNA polymerase II, thereby enhancing gene expression and contributing to tumorigenesis [78] (Figure 4B). Another study indicates that SMFQ undergoes LLPS via prion-like domains and recruits Smad4 to inhibit the formation of the Smad complex, thereby attenuating the growth inhibition response to TGF-β signaling and driving tumor growth [79].

A notable spatial-functional paradox is exemplified by USP39 [16]. USP39 preferentially partitions into the nucleolus, the canonical site of ribosome biogenesis, yet it drives upregulation of GLI1, a transcription factor that functions within the nucleoplasm. Two non-mutually exclusive mechanistic models have been proposed to bridge these two subnuclear compartments. One model posits that the nucleolus acts as a dynamic sequestration or storage hub for USP39; altered LLPS may release USP39 from nucleolar reservoirs to modulate downstream events in the nucleoplasm. An alternative model suggests that USP39 phase separation reshapes global splicing processing, indirectly increasing the abundance of functional GLI1 transcripts and protein in the nucleoplasm.

These oncogenic mechanisms provide new insights into the etiology of cancer and highlight how aberrant LLPS can drive oncogenic transcriptional programs.

**Figure 5:**
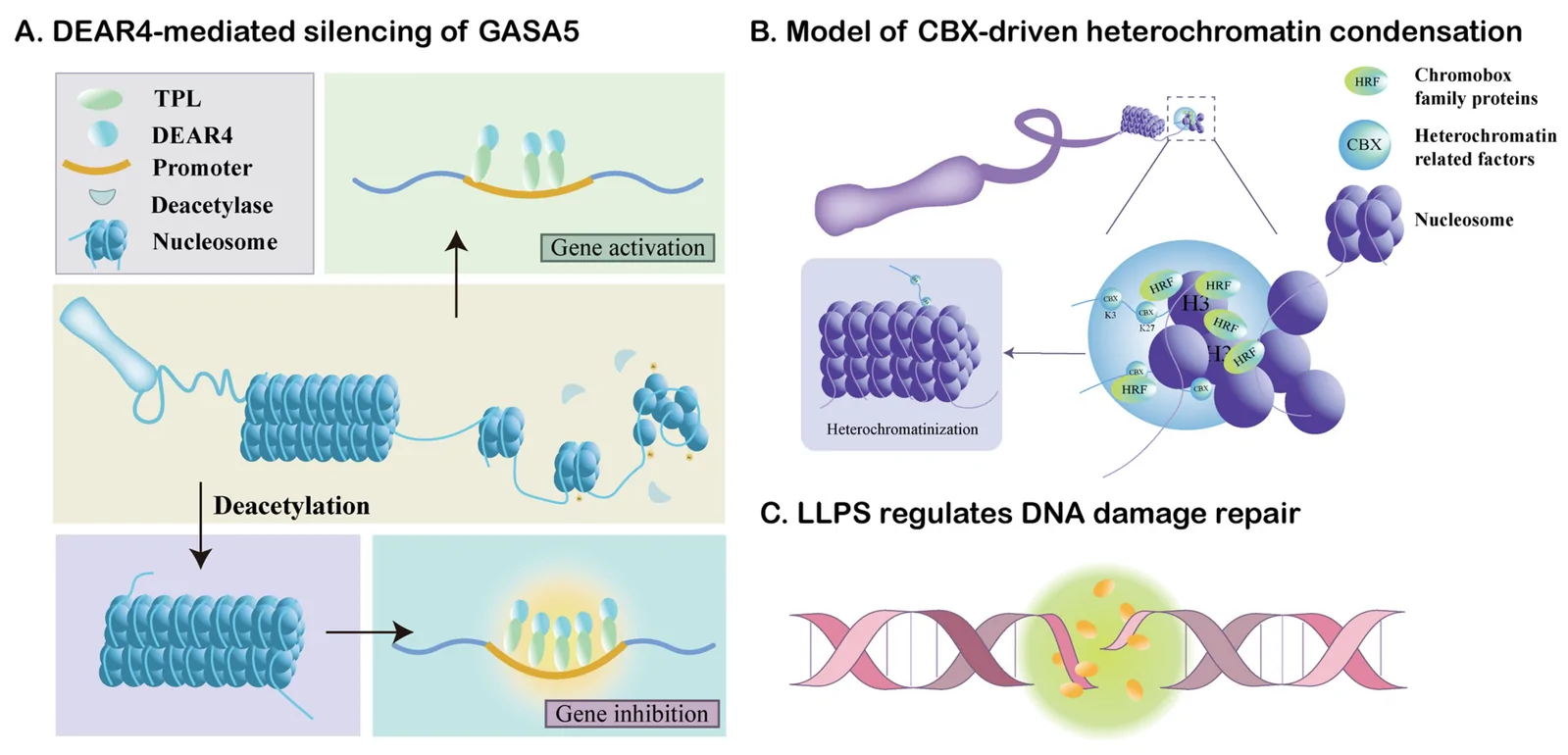
LLPS-mediated mechanisms underlying transcriptional regulation and DNA damage repair: (**A**). Schematic diagram illustrating the molecular mechanism by which DEAR4 represses the expression of GASA5. Under basal conditions, the chromatin region harboring the GASA5 gene exhibits an open configuration due to high levels of histone acetylation. The transcriptional repressor DEAR4 binds to the GASA5 promoter and, through the EAR motif located within its IDR, interacts with the corepressor TPL. This interaction promotes the formation of a transcriptional repressive condensate with phase-separation properties. The condensate enriches histone deacetylases (HDACs), which catalyze local histone deacetylation, leading to chromatin compaction and subsequent transcriptional silencing of GASA5. (**B**). CBX proteins recognize and bind histone H3 via their chromodomain. This interaction, coupled with the IDR of CBX proteins, mediates multivalent interactions that promote LLPS. The resulting biomolecular condensates selectively enrich heterochromatin factors while excluding transcriptional activators, leading to chromatin compaction and stable gene silencing. (**C**). Diagram of DNA damage repair mechanism. Upon DNA damage, key DDR proteins, such as FUS and MRNIP, undergo LLPS at lesion sites, leading to the formation of dynamic biomolecular condensates. These condensates function as repair hubs by selectively enriching DNA repair factors while excluding inhibitory proteins. This spatial compartmentalization creates a localized high-concentration microenvironment that enhances the efficiency and specificity of DNA repair processes, ensuring rapid and accurate restoration of genomic integrity.

Conversely, LLPS also recruits co-repressor factors to achieve transcriptional inhibition [80]. Experiments have demonstrated that DEAR4 interacts with the co-repressor TPL through the EAR motif within the IDR to form condensates that directly bind to the GASA5 promoter, leading to histone deacetylation and subsequent gene silencing [80] (Figure 5A). Compared with enhancers, which have been extensively studied, the mechanisms underlying silencers known as inhibitory cis-acting elements remain poorly understood.

However, reports indicate that chromobox (CBX) proteins undergo LLPS to form condensates that recruit heterochromatin-related factors such as SGO1, MeCP2, and SAFB [81,82,83], thereby contributing to the formation and maintenance of facultative heterochromatin. These condensates achieve chromatin compaction and transcriptional silencing by selectively enriching heterochromatin-related factors while excluding transcriptional activators like GTF2B [84] (Figure 5B). CBX2, through LLPS [85], specifically recognizes and binds to H3K27me3-labeled chromatin regions, facilitating the targeting of the cPRC1 complex to specific chromatin areas, promoting chromatin compaction and heterochromatin formation, and ultimately achieving transcriptional silencing [86]. The detailed review of the features of constitutively silenced heterochromatin—particularly pericentric heterochromatin, which can topologically regulate the genome [87,88]—is beyond the scope of this review.

Despite current research having revealed important insights into LLPS-mediated transcriptional regulation, several critical questions remain unresolved. The dynamic assembly and disassembly of transcriptional condensates, the mechanisms governing their spatiotemporal targeting to specific genomic loci, and the existence of “super-silencers” analogous to super-enhancers are areas requiring further investigation. Additionally, understanding how silencing complexes exclude transcriptional activators and whether these processes can be therapeutically modulated will be crucial for developing interventions targeting diseases associated with aberrant LLPS. Addressing these issues will not only deepen our understanding of LLPS in gene regulation but also open new avenues for treating diseases linked to aberrant condensate formation.

### 3.2. LLPS in DNA Repair Processes

LLPS plays a pivotal role in orchestrating the DNA damage response (DDR), a critical process for maintaining genomic integrity. Unrepaired DNA damage, particularly DNA double-strand breaks (DSBs), can lead to genomic instability [89], a defining feature of cancer [90], via the activation of proto-oncogenes and inactivation of tumor suppressor genes [89], thereby disrupting epigenetic mechanisms such as DNA methylation and histone acetylation [91].

Following DNA damage, the key proteins involved undergo LLPS to form biomolecular condensates at the sites of damage, thereby facilitating efficient DNA repair (Figure 5C), which is essential for maintaining genomic integrity [92]. For instance, DNA/RNA-binding protein FUS undergoes LLPS to promote the recruitment of critical DDR factors like SFPQ, initiating the DDR cascade [93]. Additionally, the MRN complex-interacting proteins (MRNIP), recognized as a novel cross-protective factor, form condensates that translocate to DSB sites, assisting in homologous recombination repair and activating DDR signaling [94,95].

However, cancer cells can hijack this central DDR mechanism to acquire therapy resistance. Radiotherapy resistance, a major cause of treatment failure, is often driven by aberrantly accelerated DDR [96]. Concurrently, DDR factors accumulate at DNA damage sites, thereby creating an efficient microenvironment that accelerates the DDR process [93,94]. This maintenance of genomic stability, achieved through DNA repair or apoptosis induction, can mitigate radiotherapy resistance [97]. While the tumor suppressor p53 is typically activated upon DNA damage to trigger cell death or arrest, certain proteins exploit LLPS to subvert this response [98]. For instance, the nucleolar protein NOP53 relies on its IDR1 to undergo LLPS in the cell nucleus, inhibiting the p53 pathway and enhancing radiotherapy resistance in colorectal cancer (CRC). Notably, high NOP53 expression is associated with poor radiotherapy responses in CRC patients [97], indicating that NOP53 may serve as an effective therapeutic target for these patients. Similarly, the protein NONO forms nuclear condensates through LLPS to recruit epidermal growth factor receptor (EGFR) and DNA-dependent protein kinase (DNA-PK), enhancing their interaction and promoting DNA-PK phosphorylation at T2609, thereby accelerating DDR-mediated radiotherapy resistance [99]. Collectively, these findings highlight the critical role of LLPS in regulating tumor radiotherapy resistance by modulating DDR and p53 signaling. Targeted strategies for DNA repair have great potential for cancer treatment, and the development of LLPS inhibitors targeting relevant molecular mechanisms to enhance the radiosensitivity of tumor cells warrants further investigation [97,99].

LLPS plays a critical role in DNA repair. The formation of phase-separated condensates enables targeting to DNA damage sites and the selective enrichment of key DDR factors, thereby improving the efficiency of DNA repair. This process drives the assembly of DDR factors and signal transduction, supporting a rapid response and efficient repair of DNA damage [100]. However, dysregulated DNA repair complexes or mutations that disrupt LLPS may lead to impaired DNA repair function, thereby triggering radiotherapy resistance and increasing the risk of cancer treatment failure. Moreover, if DNA damage is not repaired in a timely manner, genomic stability is severely compromised, which can further drive tumorigenesis [93,101]. Therefore, a comprehensive understanding of the molecular mechanisms by which LLPS regulates DNA repair is of great significance for maintaining genomic stability and developing novel therapeutic strategies for cancer.

In summary, LLPS-mediated condensate formation is crucial for targeting DNA damage and concentrating DDR factors to improve DNA repair efficiency. Dysregulation of this process, such as the formation of abnormal repair complexes and relevant mutations, can lead to radiotherapy resistance and increase the risk of cancer treatment failure. Understanding the molecular underpinnings of LLPS in DNA repair is therefore crucial for developing strategies to maintain genomic stability and overcome therapeutic resistance.

### 3.3. LLPS in Cellular Metabolic Reprogramming

LLPS has emerged as a pivotal mechanism that orchestrates metabolic reprogramming, a well-established hallmark of cancer [102]. Its interplay with metabolic reprogramming is increasingly understood to play a critical role in tumor progression and adaptation. Metabolic reprogramming enables cancer cells to remodel core metabolic pathways, such as glycolysis and amino acid metabolism, to satisfy the biosynthetic and energetic demands driving rapid proliferation and survival under stress. This process may result in the aggregation of certain enzymes or metabolites, thereby promoting the occurrence of LLPS [103]. Growing evidence indicates that this rewiring of cellular metabolism can promote the formation of biomolecular condensates via LLPS, while in a reciprocal manner, phase-separated compartments can spatially organize metabolic enzymes and pathways to enhance their efficiency and specificity.

The relationship between metabolic reprogramming and LLPS operates through several interconnected mechanisms. Many metabolic enzymes possess IDRs or low-complexity domains that facilitate multivalent interactions, driving their condensation into membraneless organelles when local metabolite concentrations rise. For instance, under nutrient stress, glutamine is essential for the normal growth and proliferation of cancer cells [104]. Under conditions of nutritional deficiency, cancer cells can obtain glutamine by breaking down biological macromolecules [105]. The lncRNA *GIRGL* promotes the LLPS of CAPRIN1 with glutaminase 1 (GLS1) mRNA, repressing GLS1 translation and allowing cancer cells to adapt to glutamine scarcity. Through metabolic reprogramming, cancer cells can survive in glutamine-deprived conditions [106]. This demonstrates how metabolic stress cues can directly harness LLPS to fine-tune enzyme expression. Conversely, LLPS-formed condensates create microenvironments that concentrate metabolic enzymes and substrates, thereby accelerating reaction rates and channeling metabolic flux. In CRC, the protein GPS2 undergoes LLPS to reorganize the LATS1-YAP signaling axis, leading to lipid metabolism reprogramming that fuels tumor growth [107]. Similarly, glycogen accumulation can induce LLPS and inactivate the Hippo pathway, activating YAP-driven oncogenic programs [108]. Enhanced purine synthesis via purinosome formation provides another example of how LLPS enhances anabolic metabolism in cancer [109].

Targeting LLPS and metabolic reprogramming holds significant therapeutic potential. Disrupting oncogenic condensates—such as those formed by GIRGL-CAPRIN1 or GPS2—could suppress adaptive metabolic pathways that tumors rely on under stress. However, clinical translation faces challenges, including achieving selective disruption of cancer-specific condensates without impairing essential physiological processes, and overcoming compensatory mechanisms that may lead to drug resistance. Future research should focus on mapping the complete network of interactions between metabolic fluxes and LLPS dynamics, identifying key regulatory nodes amenable to intervention, and developing compounds that can modulate the material states of pathologic condensates.

The crosstalk between LLPS and metabolic reprogramming represents a pivotal axis in cancer biology. Metabolic alterations drive the formation of specialized biomolecular condensates that, in turn, optimize metabolic pathways for tumor survival and growth. Deciphering the molecular mechanisms underlying this reciprocal relationship will not only deepen our understanding of tumorigenesis but also pave the way for novel therapeutic strategies aimed at disrupting this synergy to impair cancer progression.

### 3.4. LLPS in Stress Granule-Associated Cancer Biology

SGs are classic membraneless organelles formed through LLPS. They are composed of RNA and RBPs, assembling through multiple mechanisms in response to diverse cellular stresses [110], such as oxidative stress, nutrient deprivation, viral infection, and environmental challenges [111]. SGs serve as adaptive hubs that modulate mRNA translation, signaling cascades, and cell survival. Their dysregulation has been implicated in diverse diseases, among which are cancer and neurodegeneration [112,113].

SGs contain a variety of biomolecules, including translation initiation factors, ATPases, and RBPs harboring IDRs [114]. Among these, IDRs are indispensable for SG assembly, enabling multivalent interactions that drive LLPS [115]. Core SG scaffolds such as G3BP1 and G3BP2 not only promote condensation but also contribute to cancer proliferation, invasion, and metastasis [48,116]. Additionally, the assembly of SGs can protect tumor cell survival and suppress senescence [117]. It is noteworthy that SGs formed through different pathways may exhibit functional differences [116]. The crosstalk between SGs and cancer metabolism is tightly regulated by key signaling pathways, among which the mechanistic target of rapamycin (mTOR) pathway plays a pivotal role [48,116]. mTOR exists as two essential complexes: mTORC1 and mTORC2 [118]. Activation of mTORC1 modulates SG assembly through intricate downstream mechanisms [119,120]. A growing body of evidence links SG biogenesis to the response of cancer cells to clinical anticancer therapies, including chemotherapy and radiotherapy, though critical mechanistic details remain to be elucidated. For chemotherapy, multiple agents have been shown to induce SG assembly through distinct pathways: sorafenib, a receptor tyrosine kinase (RTK) inhibitor, and the combination of cisplatin with thapsigargin drive SG formation via PERK-dependent eIF2α phosphorylation [121,122]; oxaliplatin upregulates the SG regulator ATXN2L to enhance condensate assembly [123]; and platinum-based drugs may induce SGs through oxidative stress [124]. However, direct evidence linking SGs to chemotherapy resistance is currently lacking, and the precise capacity of chemotherapeutic agents to directly trigger SG assembly remains an open question. For radiotherapy, an anticancer modality that induces cell death via direct DNA damage or reactive oxygen species (ROS)-mediated indirect DNA damage [125], cancer cells acquire radioresistance by assembling SGs in response to radiation exposure [126], thus establishing a clear functional link between SGs and therapeutic resistance in this context.

Given the critical role of SGs in cancer progression and therapeutic response, targeting SG biogenesis and function has emerged as a promising novel strategy for anticancer therapies [116]. Based on the molecular principles governing SG assembly, three major therapeutic approaches have been proposed [116]: (1) Inhibiting canonical SG assembly via blocking eIF2α phosphorylation. The conventional mechanism of SG assembly is dependent on eIF2α phosphorylation. Therefore, reducing its phosphorylation level effectively suppresses condensate formation. Marine sponge-derived natural metabolite Psammaplysin F directly decreases eIF2α phosphorylation levels [127], while the integrated stress response inhibitor (ISRIB), an eIF2B antagonist, indirectly inhibits the downstream signaling of eIF2α and mitigates stress-induced eIF2α phosphorylation [128,129]. Targeting eIF2α or its downstream effector eIF2B thus represents a therapeutic strategy for blocking canonical SG assembly [130]. (2) Promoting atypical SG assembly via eIF4F complex inhibition. The eIF4F complex (composed of eIF4A, eIF4E, and eIF4G) is a key mediator of translation initiation [131]. Under cellular stress, translational inhibition drives eIF4F dissociation or partial inactivation, inducing the assembly of atypical SGs, which are independent of eIF2α phosphorylation [132]. Unlike classical SGs, atypical SGs lack pro-survival components due to their distinct internal composition, ultimately inhibiting tumor cell survival [120]. Inhibiting eIF4F activity via motif-specific inhibitors to promote atypical SG assembly thus represents a novel anticancer strategy that leverages SG functional heterogeneity [133]. (3) Targeting core SG structural components and regulatory interactions. Direct targeting of core SG scaffold proteins is a third promising strategy: compound C108 specifically targets G3BP2 [134], while phosphatase inhibitors such as Okadaic acid and vanadate increase the phosphorylation level of G3BP, preventing its binding to HDAC6 and inhibiting SG assembly [135]. SG biogenesis also relies on interactions with microtubules [136], making microtubule-targeting agents viable tools for regulating SG assembly. Nocodazole and vincristine are well-characterized microtubule inhibitors for this purpose [137], although high concentrations of vincristine may paradoxically promote SG assembly [138], highlighting the need for dose-dependent optimization of such agents.

The functional heterogeneity of SGs and the dual effects of distinct SG subtypes in therapeutic resistance represent major obstacles for translational research. Dissecting the molecular details governing SG assembly and distinguishing pro- versus anti-tumor SG subtypes will advance SG-targeted strategies from basic research toward clinical application and offer new avenues for anti-tumor therapy.

## 4. Therapeutic Targeting of LLPS: Applications and Prospects

Many intrinsically disordered proteins (IDPs) lack a well-defined three-dimensional structure, rendering conventional target-based therapeutic strategies ineffective and posing a significant threat to human health. However, targeting aberrant phase-separation processes can overcome this limitation. Representative strategies include small-molecule modulators, antisense oligonucleotides (ASOs), and RNA interference (RNAi).

### 4.1. Small-Molecule Inhibitor in LLPS Therapy

The therapeutic targeting of biomolecular condensates formed through LLPS represents a promising strategy for intervening in diseases driven by proteins traditionally considered “undruggable”. By modulating the dynamic equilibrium of condensates, small molecules can alter their formation, material properties, or dissolution, offering a novel pharmacological approach to disease treatment.

In cancer, aberrant LLPS can drive oncogenic signaling. For example, mutations in the non-receptor protein tyrosine phosphatase (PTP) SHP2 encoded by the *PTPN11* gene promote an open conformation that undergoes LLPS, whereas this phenomenon is not observed in the wild-type protein [139,140]. This pathological condensation activates the RAS-MAPK signaling pathway and drives cancer progression [140]. Small-molecule inhibitors that stabilize SHP2 in its closed conformation suppress mutant LLPS, thereby providing a targeted mechanism to counteract oncogenic signaling [141,142]. Similarly, the ferroptosis suppressor protein FSP1 forms condensates upon interaction with the inhibitor icFSP1 [143]. When combined with conventional ferroptosis inducers, this promotes cell death in tumors, illustrating how modulating LLPS can synergize with existing therapies.

In neurodegenerative disorders, small molecules can prevent the liquid-to-solid transition of pathological proteins. The aminosterol Claramine stabilizes α-synuclein condensates and inhibits their aggregation into amyloid fibrils, both in vitro and in *Caenorhabditis elegans* models of PD [144]. Claramine functions by inhibiting primary nucleation within condensates, demonstrating how stabilizing the liquid phase can suppress toxicity [144]. In ALS, tankyrase, a poly(ADP-ribose) polymerase (PARP), promotes TDP-43 recruitment into SGs and cytoplasmic accumulation. Tankyrase inhibitors reduce TDP-43 pathology and mitigate neurodegeneration [145].

Drug repurposing has emerged as a valuable approach for identifying LLPS modulators. Sildenafil, a phosphodiesterase-5 inhibitor (PDE5I), reduces Aβ aggregation and tau hyperphosphorylation in Alzheimer’s models, and epidemiological data are associated with a lower incidence of AD [146,147]. Similarly, the anti-HIV drug elvitegravir disrupts LLPS-driven condensates formed by the transcriptional co-activator SRC-1 and TEAD, thereby suppressing YAP-mediated oncogenic transcription [148].

Small-molecule inhibitors can modulate pathological LLPS through diverse mechanisms. They may act as dissolvers to disrupt existing condensates, inducers to promote the formation of novel condensates with altered properties, or morphers that change material properties to prevent harmful liquid-to-solid transitions. We herein use the term condensation modification therapy (c-mods) to define condensate-targeting therapeutic agents. In affected cells, c-mods are capable of disrupting the assembly of disease-relevant biomolecular condensates and competitively binding to these condensates in a selective manner. This excludes target molecules from the condensate microenvironment. Alternatively, c-mods can suppress the normal biological functions of target molecules residing within condensates, thereby providing a robust therapeutic strategy for the intervention in cancer, neurodegenerative diseases, and multiple other condensate-associated disorders [4].

### 4.2. Selective Enrichment in LLPS Therapy

Biomolecular condensates formed via LLPS enable the dynamic assembly and disassembly of biomolecular components. This process drives the selective enrichment of specific components, thereby allowing for targeted modulation of cellular activities. A key therapeutic benefit of such condensates is that small-molecule drugs preferentially partition into specific compartments, boosting efficacy while reducing off-target effects.

Small-molecule drugs can exhibit selective enrichment within certain condensates [149]. For instance, cisplatin is highly concentrated in MED1 condensates, whereas mitoxantrone accumulates in FIB1 and NPM1 condensates [149]. This selective partitioning effectively enhances local drug concentration and efficacy. In the case of MED1-cisplatin condensates, the aromatic residues of MED1 create a physicochemical environment that maintains high fluidity of the droplet, accelerating the dissolution of MED1 condensates [149].

Protein LLPS typically involves complex molecular interactions within condensates. The protein FIP4 can target the FOXM1 condensate, bind to FOXM1, and phosphorylate Ser376, thereby disrupting FOXM1 LLPS. Studies have shown that treatment with FIP4 significantly inhibits tumor growth and proliferation in breast cancer mouse models [19]. Similarly, the transcription factor c-MYC, a representative IDP, forms a dimer with MAX to drive oncogenesis [150]. Small molecules such as IIA6B17 and IIA4B20 can disrupt the initial formation of the c-MYC-MAX dimer, thereby preventing tumor growth and progression [151].

The non-peptide small molecule R-huezole demonstrates the principle of co-condensation. It forms droplets in aqueous solutions and selectively co-condenses with the α, β, and γ subunits of tubulin. This process inhibits cell mitosis by sequestering tubulin away from centrosomes or spindle formation sites. By taking advantage of the covalent linkage between the protein ligand of R-huezole and its self-assembling moiety, we can rationally design analogous self-assembling small molecules, providing novel insights for the therapeutic development of related diseases [31,152].

Natural polyphenols are potential inhibitors of protein aggregation. Tannic acid (TA) has been shown to inhibit the aggregation of tau protein, α-synuclein, and β-amyloid [153,154]. In the presence of TA, phosphorylated tau protein dissolves within the condensates, inhibiting aggregation through LLPS [155]. Furthermore, compounds like EGCG, suramin, and L-Dopa-grafted polymers can enhance the fluidity of tau protein droplets, offering new insights for treating neurodegenerative diseases [156,157,158].

Further exploring the correlation between diseases and LLPS, as well as designing targeted drugs based on the selective enrichment property of phase separation, can effectively improve therapeutic efficacy, reduce off-target effects, and mitigate the side effects of clinical treatment. Through selective enrichment, it is feasible to remodel the properties of pathological condensates, thereby specifically blocking oncogenic pathways and offering new insights for future drug development.

### 4.3. Antisense Oligonucleotides and RNA Interference in LLPS Therapy

ASOs and RNAi represent emerging therapeutic modalities offering promise for treating diseases linked to aberrant LLPS. Both strategies rely on sequence-specific binding to complementary RNA, leading to gene silencing through distinct yet related mechanisms [159]. Recent reports indicate that the intracellular assembly and activity of ASO- and RNAi-induced complexes may rely on similar biophysical principles, including multivalent interactions and compartmentalization, to those driving biomolecular condensate formation. While direct evidence linking these nucleic-acid-based approaches to the physical mechanisms of LLPS remains limited, their ability to modulate gene expression by targeting specific RNA sequences suggests potential synergy with LLPS pathways.

In the context of neurodegenerative diseases, ASOs and RNAi strategies are being explored to indirectly modulate pathological LLPS [160,161,162]. For example, in ALS, where TDP-43 aggregates are a pathological feature, directly silencing TDP-43 is not feasible due to its essential cellular functions [24,42]. Instead, targeting upstream regulators such as ataxin-2 with ASOs has shown promise in preclinical models, slowing disease progression and reducing TDP-43 aggregation [163,164]. Similarly, in a mouse model of FUS-associated ALS, the FUS-targeting ASO ION-363 significantly suppresses FUS expression and reduces FUS protein aggregation, demonstrating the therapeutic promise of ASO-targeted approaches [165]. In cancer, these strategies can address LLPS-related mechanisms of drug resistance. For instance, in hepatocellular carcinoma (HCC), resistance to the targeted therapeutic agent sorafenib is partly mediated by the inhibition of ferroptosis [20]. The lncRNA *URB1-AS1* drives ferritin LLPS, which lowers cellular free iron levels and suppresses ferroptotic activity. Combination treatment with sorafenib and a GalNAc-conjugated siRNA targeting *URB1-AS1* has been shown to restore drug sensitivity in resistant HCC cells, illustrating how RNAi can overcome resistance mechanisms associated with biomolecular condensate biology [20]. Beyond neurodegeneration and cancer, ASOs and RNAi may also modulate transcriptional condensates implicated in disease. During macrophage polarization, the transcription factor YY1 forms a complex with coactivators including p300, p65, and CEBPB within nuclear condensates. Silencing *YY1* using si*YY1* has been shown to significantly suppress tumor growth in mouse models, suggesting that disrupting such transcriptional hubs may hold therapeutic potential [166].

ASOs and RNAi therapies have emerged as versatile and highly specific novel tools for the treatment of diseases driven by pathogenic LLPS. Such therapeutic modalities modulate the formation and assembly of phase-separated aggregates by targeting key regulatory RNAs, thereby achieving indirect therapeutic effects on these disorders. Nevertheless, current ASO and RNAi strategies still present several inherent limitations. Further elucidation of the molecular mechanisms underlying the interplay between RNAs and phase-separated condensates is therefore urgently required, and optimized therapeutic strategies need to be developed to broaden their applicability to a wider spectrum of LLPS-associated diseases.

### 4.4. RNA in LLPS Therapy

As one of the most negatively charged macromolecules [167], RNA readily interacts with RBPs to form ribonucleoprotein (RNP) condensates [168,169]. This spontaneous assembly is driven by multivalent intermolecular interactions, which predominantly occur through protein–protein, protein–RNA, and RNA–RNA contacts during LLPS [169,170]. In addition, perturbations in RNA levels or alterations in RBP-RNA binding capacity can lead to aberrant phase transitions [171]. Notably, the RNA-binding domain of RBPs is essential for LLPS, and the presence of RNA significantly reduces the concentration threshold required for RBP-driven LLPS [172,173]. Collectively, RNA acts as a crucial driver and regulator of biomolecular condensate assembly in diverse cellular contexts [174].

**Figure 6:**
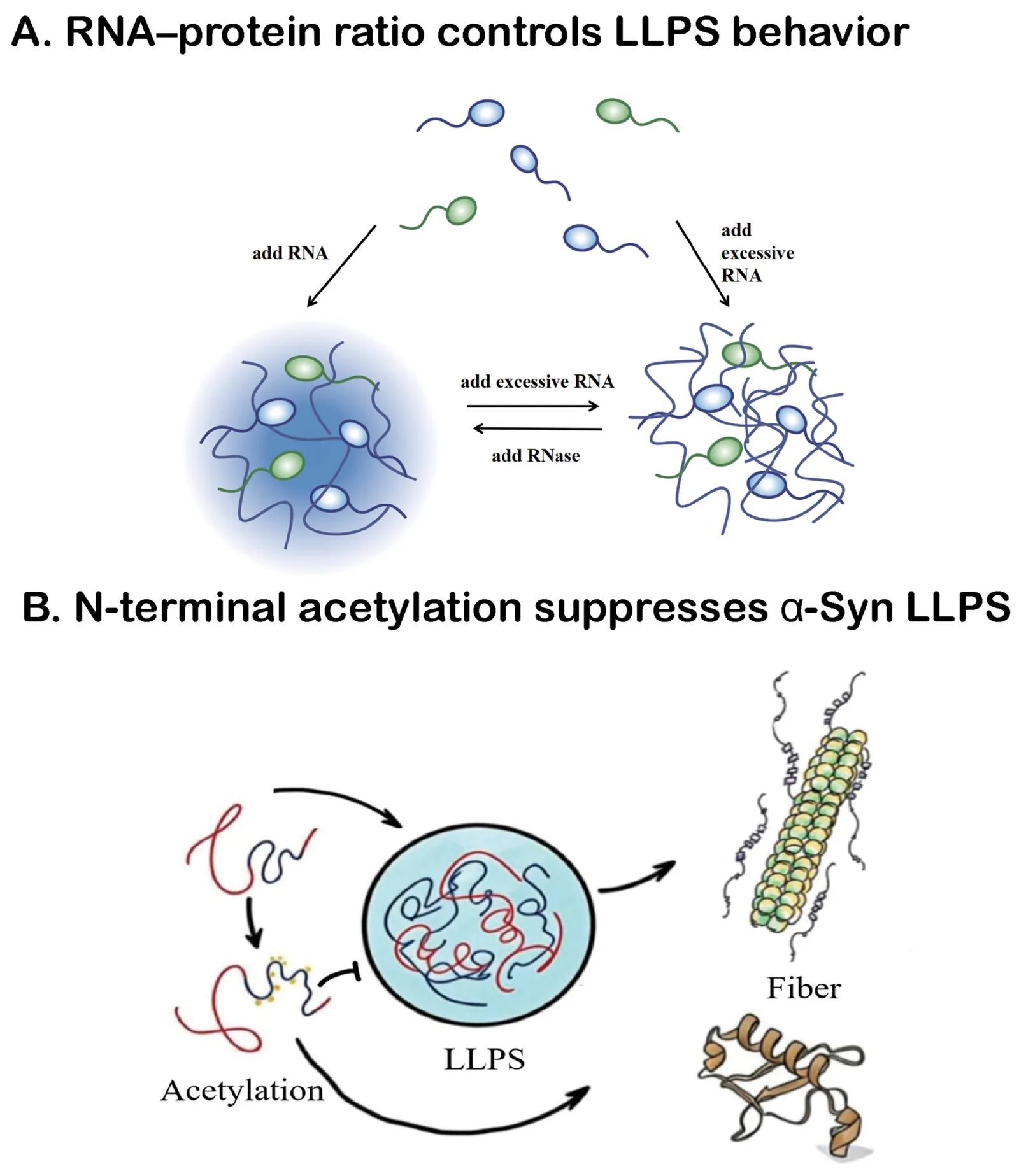
LLPS and pathological aggregation of neurodegeneration-associated proteins: (**A**). The ratio of RNA to protein (RNP) is a critical determinant of the phase behavior of biomolecular condensates. At low RNA concentrations, multivalent RNA-protein interactions drive the nucleation and growth of condensates. However, at high RNA concentrations, the system experiences a dilution effect that attenuates protein-protein contacts, leading to the dissolution of condensates. The addition of RNase A reduces RNA levels, thereby restoring a low RNA/protein ratio and re inducing LLPS. These results demonstrate that a low RNA/protein ratio promotes LLPS, whereas a high ratio inhibits condensate formation. (**B**). Molecular Mechanism of N-Terminal Acetylation-Mediated Inhibition of α-Syn LLPS. α-Syn undergoes LLPS to form biomolecular condensates, which can subsequently transition into solid amyloid fibrils. N terminal acetylation stabilizes the α helical conformation of the N terminal region and disrupts the long-range interactions between the N- and C-terminal domains. This structural modulation effectively prevents the formation of phase-separated condensates, thereby inhibiting the downstream amyloid aggregation pathway.

The binding of RNA to RBPs not only nucleates condensates but also fine-tunes their material properties and stability. For instance, tau proteins bind selectively and strongly to RNA, thereby prolonging the liquid-like state of tau condensates and slowing their conversion into pathological aggregates—a phenomenon also documented for TDP-43 [171]. These observations indicate that targeting RNA-RBP interactions may serve as a promising approach to slow disease progression in neurodegenerative disorders. Similarly, the RNA-to-protein ratio critically regulates LLPS: low ratios promote condensate formation, whereas high ratios can inhibit or even dissolve droplets, as demonstrated in studies of prion-like RBPs such as FUS, TDP-43, and hnRNPA1 [171] (Figure 6A).

LncRNAs have emerged as key architectural and regulatory components of phase-separated compartments [89]. By acting as molecular scaffolds, lncRNAs recruit multiple proteins to propel LLPS and regulate RNA processing, transcription, and decay within condensates [175,176]. For instance, lnc-*MALR* promotes the LLPS of ILF3, activating the HIF1α signaling pathway to drive esophageal squamous cell carcinoma (ESCC) progression [177]. In clear cell renal cell carcinoma (ccRCC), lnc-*MIRE* enhances the mRNA-binding affinity of heterogeneous nuclear ribonucleoprotein K (hnRNPK) and eukaryotic translation initiation factor 2 (eIF2) through LLPS, stabilizing oncogenic transcripts [175]. Conversely, several lncRNAs function as tumor suppressors. For example, GATA2-AS1 inhibits the LLPS of FUBP3 and interacts with SUZ12 to block the malate-aspartate shuttle, thus disrupting energy metabolism in neuroblastoma [178]. Similarly, lnc-*FASA* directly binds to PRDX1, promoting its LLPS, inhibiting peroxidase activity, disrupting redox homeostasis, and inducing ferroptosis in breast cancer [179].

The detailed application of RNAi in drug therapy has been extensively described in the preceding text and will therefore be omitted here.

RNA plays a pivotal role in the biophysical process of LLPS, offering profound insights into disease etiology and therapeutic intervention. Targeting this mechanism through modulation of RNA expression or the design of RBP-specific drugs represents a promising avenue for developing novel therapies. Furthermore, despite the nascent stage of current research, the intersection of RNA biology and LLPS provides unprecedented opportunities to elucidate complex pathogenic networks and establish innovative treatment paradigms.

### 4.5. Post-Translational Modification in LLPS Therapy

PTMs play a pivotal role in the targeted modulation of LLPS and represent a promising therapeutic avenue for diseases driven by aberrant condensate formation [180]. Key PTMs, including phosphorylation, acetylation, methylation, ubiquitination, and sumoylation significantly influence the propensity, dynamics, and material properties of phase-separated assemblies [181,182]. Their combinatorial action often provides a finely tuned regulatory layer over condensate behavior.

In the DDR, sumoylation of RNF168 drives its LLPS, sequestering the protein away from damaged DNA sites and impeding non-homologous end joining repair. The desumoylase SENP1 dissolves condensates and restores DDR efficiency, highlighting how reversible sumoylation can spatially control the DNA repair machinery [183]. In neurodegeneration, PTMs critically modulate the phase behavior of pathological proteins. Under physiological conditions, N-terminal acetylation of α-syn stabilizes an α-helical conformation and inhibits long-range intramolecular interactions, thereby suppressing LLPS [184] (Figure 6B). However, in disease contexts, this same modification can paradoxically promote α-syn aggregation, underscoring the context-dependent effects of PTMs and the need for condition-specific therapeutic strategies [185]. Similarly, arginine hypomethylation in FUS enhances its LLPS and gelation via cation-π interactions, while tyrosine phosphorylation inhibits condensate formation by disrupting key interaction interfaces [186]. Modulating these modifications, for instance, by promoting FUS phosphorylation or restoring arginine methylation, could prevent pathological transitions and alleviate toxicity in diseases such as ALS.

In tauopathies, lysine acetylation reduces the LLPS propensity of proteins, particularly in the presence of RNA, by neutralizing positive charges and weakening electrostatic interactions [187,188]. This suggests that acetylation-mimicking strategies may help maintain tau in a soluble, functional state. Beyond neurodegeneration, PTMs also govern chromatin organization, sumoylation of HP1α promotes the formation of larger, more stable heterochromatin droplets [189].

While manipulating PTM states offers conceptual appeal for counteracting pathogenic LLPS, important pharmacological limitations must be acknowledged. Direct pharmacological targeting of broad upstream kinases or phosphatases to remodel the PTM landscape of disease-relevant proteins carries substantial risks of widespread systemic cellular toxicity. Many of these enzymes act on a large set of essential homeostatic substrates, including core DNA-damage-repair and housekeeping signaling pathways. Therefore, global perturbation of such enzymes is generally neither selective nor clinically viable for selectively suppressing pathological protein aggregation. This toxicity boundary tempers enthusiasm for simple therapeutic strategies that target LLPS-related phase behavior via wholesale modulation of upstream PTM regulators, and highlights the challenges of drugging phase-separation-driven pathology in wild-type protein backgrounds. Novel, more selective targeting paradigms are therefore required to translate PTM-dependent LLPS-modulating concepts into safe clinical interventions [190].

In conclusion, PTMs serve as essential biochemical switches that regulate the formation, dissolution, and transformation of biomolecular condensates. Targeting these modifications presents a promising, yet practically challenging, avenue for therapeutic intervention.

### 4.6. Molecular Chaperone in LLPS Therapy

Molecular chaperones represent a promising class of modulators for counteracting pathological LLPS [180]. Under physiological conditions, molecular chaperones facilitate protein folding, prevent aggregation, and maintain proteostasis. IDRs, which are prevalent in proteins associated with signaling and disease, often interact with chaperones to adopt ordered conformations or remain solubilized, thereby preventing aberrant self-assembly [191,192,193].

Beyond their canonical folding functions, certain molecular chaperones exhibit disaggregase or dissolution activity, enabling them to actively reverse pathological LLPS [194,195]. For example, the nuclear import receptor TNPO1 acts as a chaperone for FUS. While TNPO1 does not alter the methylation state of FUS, a key determinant of its phase behavior, it interacts directly with FUS, inhibiting its LLPS and gelation, and promoting the assembly and stability of ribonucleoprotein complexes [186]. Similarly, Kapβ2, another nuclear transport receptor, binds to PY-NLS motifs in proteins such as FUS and TDP-43, preventing and even reversing their aggregation in disease-related mutants [196].

Certain chaperones can dissociate proteins from solid-like aggregates through disaggregase activity, which represents an additional mechanism to combat irreversible pathological condensation [194,195]. Harnessing this property, either by enhancing endogenous chaperone function or by developing small-molecule chaperone mimics, could provide a viable strategy to dissolve pre-formed condensates and mitigate toxicity in neurodegenerative and other protein aggregation diseases.

Molecular chaperones serve as natural guardians against aberrant LLPS by facilitating proper folding, inhibiting condensate formation, and promoting disaggregation. Developing peptides that enhance or mimic these activities—particularly those of transport receptors and disaggregases—holds considerable therapeutic potential for diseases driven by pathological LLPS, such as amyotrophic lateral sclerosis, frontotemporal dementia, and related disorders. Future research should focus on identifying specific chaperone-condensate interactions, elucidating structural mechanisms of condensate dissolution, and translating these insights into targeted molecular interventions [197,198,199].

## 5. Conclusions and Outlook of LLPS

Biomacromolecules drive the assembly of biomolecular condensates via multivalent intermolecular interactions. Aberrant phase separation can contribute to the pathogenesis of a broad range of human diseases, including cancer, neurodegenerative disorders and autoimmune diseases, and can also induce the development of drug resistance.

In this review, we briefly introduce the fundamental concept of LLPS, elaborate on its close association with diverse human diseases, and summarize the latest advances in LLPS-based therapeutic strategies. Although LLPS has been well established to exert crucial functions in multiple biological processes, this research field is still in an early stage of development, and the underlying molecular mechanisms remain to be further elucidated.

In summary, translating mechanistic insights on LLPS into clinical interventions represents a rapidly evolving frontier [97,103,200,201]. Although traditional modalities like endocrine therapy and radiotherapy are currently employed, novel approaches leveraging small-molecule inhibitors, RNA silencing, and chaperone-assisted clearance have demonstrated significant efficacy in targeting pathological LLPS. These strategies not only provide practical feasibility for disease treatment but also open new avenues for understanding the biological consequences of protein condensation. Further dissection of LLPS regulatory networks will therefore be essential to fully realize the therapeutic potential of targeting aberrant phase transitions in human disease.

## Figures and Tables

**Table 1:** Proteins primarily implicated in neurodegenerative diseases and their pathological etiologies.

Proteins	Diseases	Causes of Abnormal LLPS	Therapeutic Strategy	References
Tau	AD PSP CBD AGD PiD FTD CTE	Phosphorylation	Block liquid-to-solid transition of condensates	[30,31]
FUS	ALS FTD	Pathological mutation	Modulate phase separation	[31,32]
TDP-43	ALS FTD AD PD G-PDC	Pathological mutation Phosphorylation Ubiquitination	Inhibiting phosphorylation, ubiquitination or targeting TDP-43 condensates to prevent aggregation	[33,34,35]
α-syn	LBDs PD MSA Synucleinopathies	Pathological mutation	Targeting α-syn condensates to prevent aggregation	[36,37]
Aβ (or APP)	AD	The clearance ability of Aβ is impaired	Inhibiting Aβ condensate formation	[38,39]
HTT	HD	The CAG repeat sequence within exon 1 of the HTT gene is expanded	Targeting HTT condensates to prevent aggregation	[40]

**Note.** Progressive supranuclear palsy (PSP), corticobasal degeneration (CBD), argyrophilic grain disease (AGD), Pick’s disease (PiD), some forms of frontotemporal dementia (FTD), chronic traumatic encephalopathy (CTE), Amyotrophic Lateral Sclerosis (ALS), Alzheimer’s Disease (AD), Parkinson’s disease (PD), guamanian amyotrophic lateral sclerosis/parkinsonism-dementia complex (G-PDC), Lewy Body Diseases (LBDs), multiple system atrophy (MSA) and Huntington’s Disease (HD).

**Table 2:** Examples of LLPS in immune evasion, viral replication, and autoimmune dysregulation.

Pathogen/Disease	LLPS-Associated Molecules	Biological Effect	Reference
*SARS*-CoV-2	Viral nucleocapsid (N) protein, host G3BP1	Disrupts cGAS-DNA condensates to suppress IFN response	[68]
Enteropathogenic *E. coli*	Bacterial NleE, host ZRP1	Impairs ZRP1 LLPS to disrupt host unfolded protein response	[68]
*HIV*-1	Viral integrase, host LEDGF/p75	Forms condensates enhancing viral DNA integration	[70]
SLE	Host USP8, DDX3X, cGAS	Promotes cGAS activation via DDX3X deubiquitination	[55,56]
RA	*MYC*, *MAP1LC3B*	Gene expression changes suggest LLPS involvement in inflammation	[54]

## Data Availability

No new data were created or analyzed in this study. Data sharing is not applicable to this article.

## Abbreviations

AD	Alzheimer’s Disease
ALS	Amyotrophic Lateral Sclerosis
ASOs	Antisense oligonucleotides
BCR	B-cell receptor
cGAMP	Cyclic GMP-AMP
cGAS	Cyclic GMP–AMP synthase
CBX	Chromobox protein
CRC	Colorectal cancer
CVDs	Cardiovascular disease
DDR	DNA damage response
DSBs	DNA double-strand breaks
IDRs	Intrinsically disordered regions
LATE	Limbic-predominant age-related TDP-43 encephalopathy
LLPS	Liquid–liquid phase separation
lncRNA	Long non-coding RNAs
MAZ	Myc-associated zinc finger protein
PTMs	Post-translational modifications
RA	Rheumatoid arthritis
RBPs	RNA-binding proteins
RNAi	RNA interference
RNP	Ribonucleoprotein
SGs	Stress granules
STING	Stimulator of interferon genes
TCRs	T-cell receptors
XCI	X chromosome inactivation
